# Supplementary material for: S100B Protein as a Therapeutic Target in Multiple Sclerosis: The S100B Inhibitor Arundic Acid Protects from Chronic Experimental Autoimmune Encephalomyelitis
Source: Int J Mol Sci. 2021 Dec 17;22(24):13558. doi: 10.3390/ijms222413558 (PMC8708367; doi:10.3390/ijms222413558)
Supplement: Supplementary file 1 [file ijms-22-13558-s001.zip › ijms-1495130-supplementary.pdf]

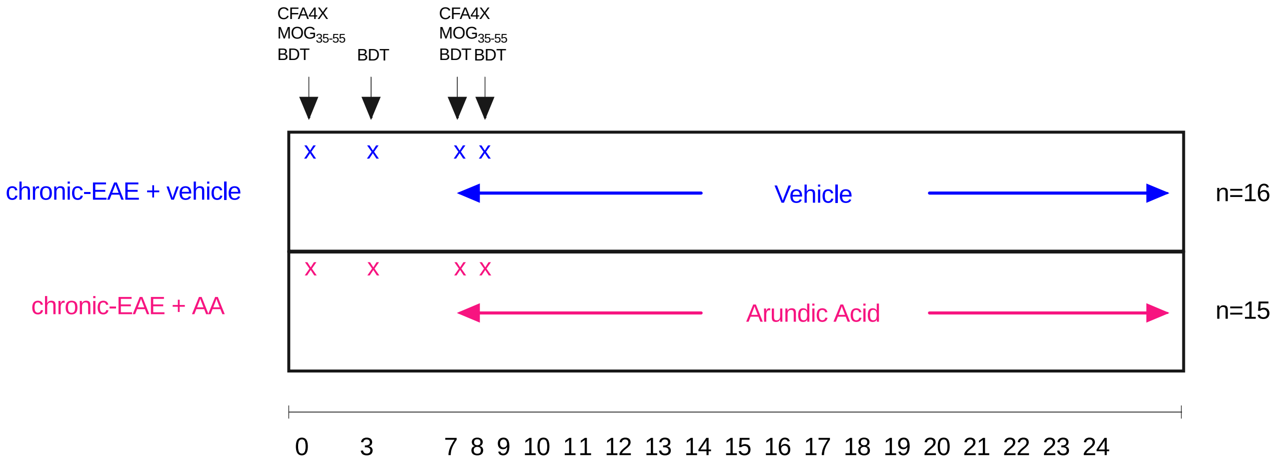

Supplementary Figure S1: Timetable for experimental procedure of chronic EAE: EAE was induced in C57Bl/6 mice subcutaneously administering at d0 and an emulsion composed by CFA (4 times concentrated) and $MOG_{35\text{-}55}$. The Bordetella Pertussis toxin was intraperitoneally administered at d0, d3, d7 and d8. The arundic acid treatment began on d7 and was delivered daily until d24 at a dosage of 4mg/kg

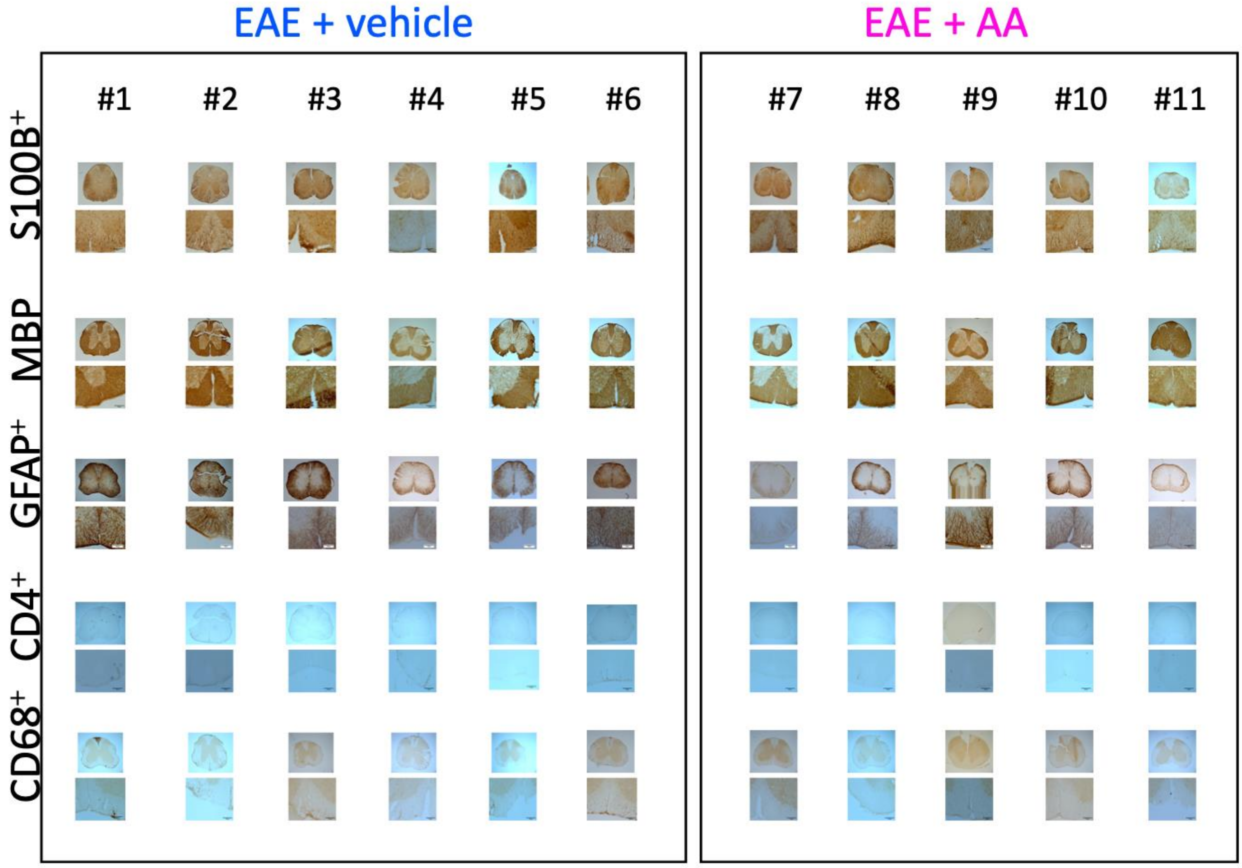

Supplementary Figure S2. Immunostaining of all C1-C4 representative images of spinal cords derived from EAE affected mice treated with vehicle (left box) and AA (right box). From each treatment groups are reported EAE mice numbers. On the vertical left side, are reported all specific immunostaining markers taken in our analysis (S100B⁺, MBP, GFAP⁺, CD4⁺ and CD68⁺). Images are reported following this order: the first lines of each immunolabeling, report the C1-C4 spinal cord images with 5 magnification (5x) and, immediately below these, their relative partial slices areas images with 20 magnification (20x). Not all the images here reported are used for our IHC semi-quantification analysis.

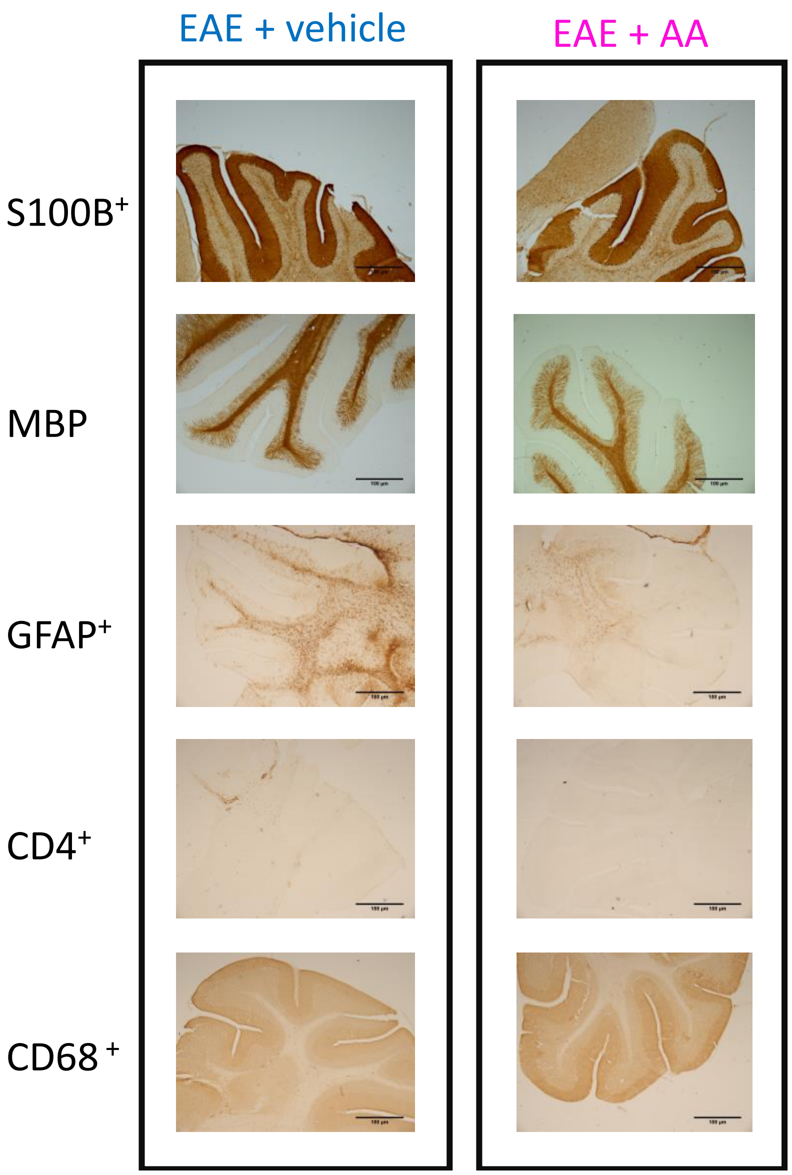

Supplementary Figure S3. Immunostaining of representative images of cerebellum areas derived from EAE affected mice treated with vehicle (left box) and AA (right box). On the vertical left side, are reported all specific immunostaining markers taken in our analysis (S100B $^{+}$, MBP, GFAP $^{+}$, CD4 $^{+}$ and CD68 $^{+}$). Images are reported following this order: the first lines of each immunolabeling, report the cerebellar images with 5 magnification (5x). The images here reported were not used for IHC semi-quantification analysis.

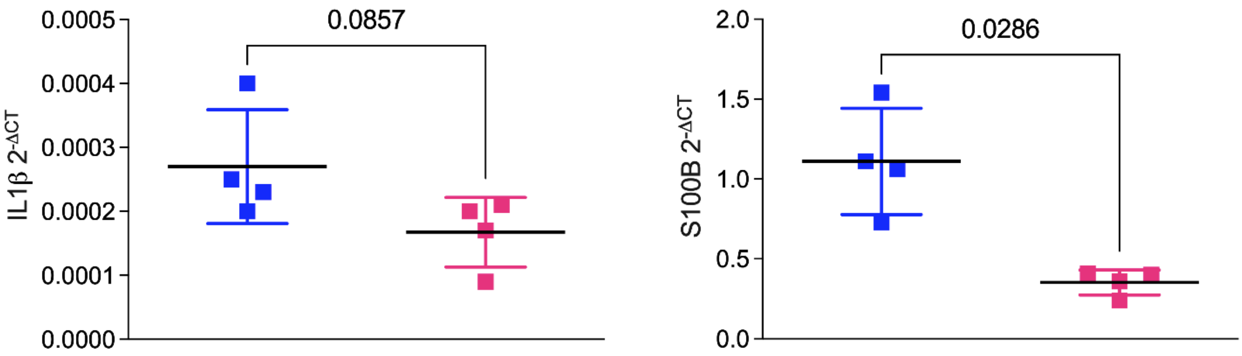

Supplementary Figure S4. Effect of Arundic Acid (AA) on IL1β and S100B from spinal cord. Comparison of gene expression levels (2^delta-cT) of IL1β and S100B between treated (AA, fuchsia squares) and untreated (vehicle, blue squares) EAE affected mice. Figure 3A: the reduction of IL1β in EAE affected mice treated with AA is not significant (p=0,0857, t-test, Mann-Whitney). S100B expression levels present significant differences between AA- and vehicle treated EAE groups (p=0,0286, t-test, Mann-Whitney).

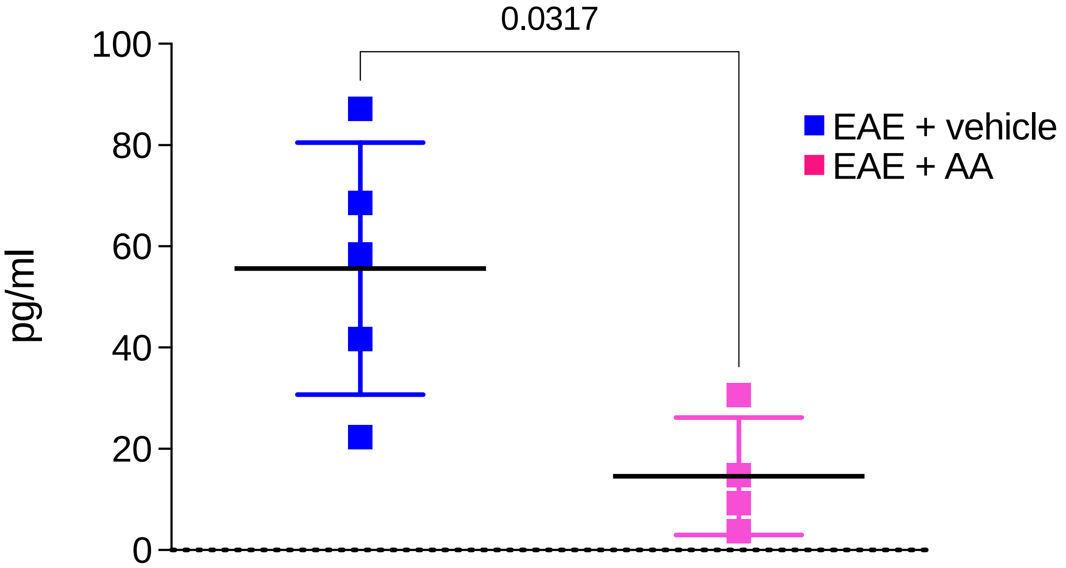

Supplementary Figure S5. Modulation by Arundic Acid of S100B astrocytic expression and serum secretion. The ELISA assay (Abcam) measured S100B levels in the sera of AA- or vehicle treated and EAE affected mice. Sera were collected from the heart of mice at the moment of sacrifice just before the perfusion procedures (24 days after immunization). The AA-treatment in EAE affected mice is able to significantly decrease the S100B levels in comparison with EAE mice treated with vehicle, reaching similar levels of S100B found in the sera from healthy mice. (p=0.0317, t test, Mann Whitney).

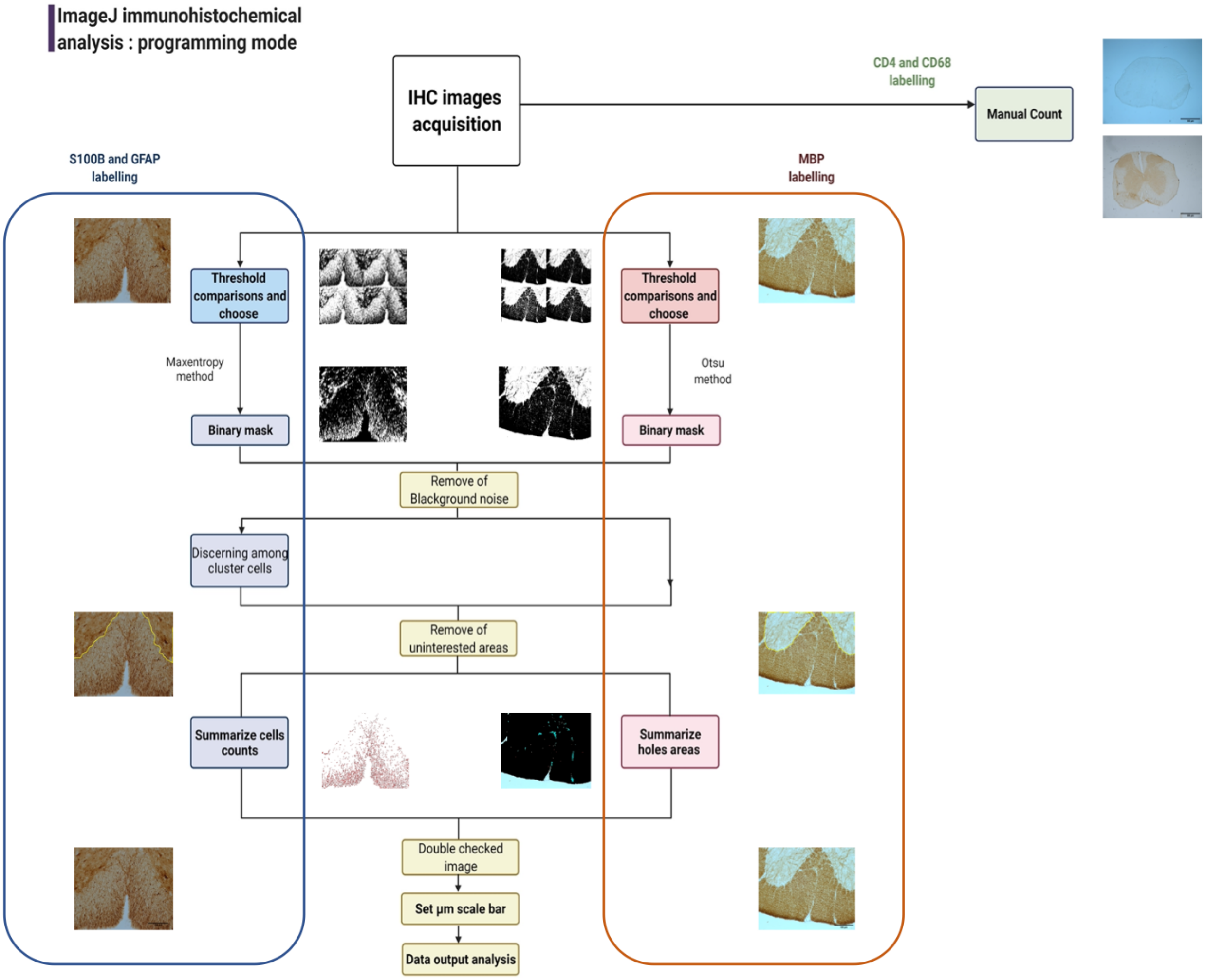

Supplementary Figure S6. Workflow of ImageJ immunohistochemical analysis. This image reports all ImageJ programming steps that have been done for IHC semi-quantification. S100B and GFAP labellings cells counts as well as for the MBP negative areas sum have been performed through unshared programming steps (blue box, left side; red box, right side) and shared steps ones (yellow box, center). CD4 and CD68 labelling has been analyzed through manual count. Diagram shows the digital logic order through images have been processed from acquisition step to the data output analysis. With images thresholds applications (Image->Adjust->Autothreshold->operator choose) and comparisons, operator has the possibility to choose the best suitable ones (in our analysis we use *Maxentropy* threshold whereas *Otsu* one for S100B/GFAP and MBP labelling, respectively). The resulting binary mask, assigning two color value (black=0; white=255), allows a further processing of images. After the background noise remove (process->noise->"remove outliers"), only S100B/GFAP clusters cells reported in IHC slides images, are additionally processed through ImageJ «watershed» command to singly discerning cells. Before carrying out the counting performance (summerize cells counts/ holes areas: "Analyze Particles", "size=0-Infinity pixel show=Outlines summarize"), operator acts to remove gray matter (uninterested areas) through free-hand ImageJ tool. In the end of the programming method, has been done a control step ("Multiply create", "original", "processed") to check what the script actually done through overlaying of the original image with the one resulting from the counting process. Therefore, to images have been applied µm scale bar ("Set Scale", "distance=; known=; unit=micron") and their relative data counting output are then analyzed. All processed images have been obtained through foorloop mode of script and singly saved in arbitrary operator directory (.tif extension files). This diagram is obtained by Biorender® software.

Supplementary Table S1. Criteria of EAE disease score. The induction of EAE was performed by an operator (GDS) who divided mice into treated and control groups, assigning different codes, dividing them into different cages and distinguishing animals with ear holes. This operator was the only one in charge of the daily treatments, of the daily registration of the scores and of the supervision of both experiments. Clinical disease score was assessed by 3 operators (CC, MDC and MT), who performed alone the double-blind examinations of the animals, ignoring both codes and treatments and the score of the other operators. The clinical assessment was performed at the same hours twice everyday (8.30-9.00 a.m. and 3.00-3.30 p.m.) except for Saturday and Sunday where it was performed only in the morning. The operators rotated each day so that the one who had visited one day in the morning would have visit in the afternoon shift of the next day and vice-versa, to avoid any bias depending on the circadian rhythm of the animal and on the routine of the visits. All the operators were trained by GDS in previous experiments and had at least 1 year experience with EAE affected mice handling. As described in the table inserted in the Supplementary Figure 1 (and reported below), a 0 to 3.5 score was used, according to the animal welfare of Health Ministry of Italy that impose a termination criterium when clinical disease score is more severe than score 3. In detail the scores are as following: 0 = healthy; 0.5 = weak tail or unsteady gait; 1 = Limp tail or weak tail and hindleg weakness/unsteady gait; 1.5 = Unilateral hindlimb weakness; 2 = Bilateral hindlimb weakness; 2.5 = Unilateral hindlimb paraplegia; 3 = Bilateral hindlimb paraplegia; 3.5 = Moderate loss of lower body control. The score resulted from the mean calculated by the operator in charge of registration of data (GDS) based on the scores assessed by the different visiting operators for each day, except for the weekend, when the evaluation was performed just in the morning.

score	Criteria
**0.5**	**Weak tail or unsteady gait**<br>(weak tonus or half of tail drops or mouse walks on the bench like a duck)
**1**	**Limp tail or weak tail and hindleg weakness/unsteady gait**<br>(tail drops or weak tail and mouse walks on the bench like a duck)
**1.5**	**Unilateral hindlimb weakness**<br>(mouse walks on the grid of the cage and one leg falls into the gaps or combination of previous scores)
**2**	**Bilateral hindlimb weakness**<br>(mouse walks on the grid of the cage and one leg falls into the gaps; mouse walks on the bench and one hindlimb is not moving; or combination of previous scores)
**2.5**	**Unilateral hindlimb paraplegia**<br>(mouse walks on the grid of the cage and one hindlimb is always in the gaps; mouse walks on the bench and one hindlimb is not moving)
**3**	**Bilateral hindlimb paraplegia**<br>(mouse walks on the grid of the cage and both hindlimbs are always in the gaps; mouse walks on the bench and is not moving its hindlimbs; lower body control has to be checked)
**3.5**	**Moderate loss of lower body control**<br>(mouse is turned to the dorsum to see if can poorly turn back; mouse falls to one side but can still move in the cage with the frontlegs)

Supplementary Table S2. Primer sequences

gene	FORWARD	REVERSE	CITATION
β-actin	5'- CGTAAAGACCTCTATGCCAACA -3'	5'-GGAGGAGCAATGATCTTGATCT-3'	[55]
Interferon γ	5'-ATGAACGCTACACACTGCATC-3'	5'-CCATCCTTTTGCCAGTTCCTC-3'	[56]
Interleukin1β	5'-TGCCACCTTTTGACAGTGATG-3'	5'-ATGTGCTGCTGCGAGATTTG -3'	[57]
S100B	5'-AAAGGCTCATGGGCTCGAAG-3'	5'-GAAGGGGGTTGGGGTTTCAT-3'	[13]
